# Parent–Child Separation Always Comes with a Cost

**DOI:** 10.3390/ejihpe13080101

**Published:** 2023-07-28

**Authors:** Luca Cerniglia

**Affiliations:** Faculty of Psychology, International Telematic University Uninettuno, Corso Vittorio Emanuele II, 39, 00186 Rome, Italy; luca.cerniglia@uninettunouniversity.net; Tel.: +39-066920761

In some situations, due to the risk of contagion, the recent COVID-19 pandemic forced parent–child separation to occur in attempts to slow down the spread of the virus. In many countries, governments’ policies recommended (or imposed) physical distance between people, even in the same family if one family member was positive to the virus and the other members were not.

In some (not so rare) cases, children have been kept distant from their parents and siblings within the same house or in different locations for long periods of time [1]. Although these measures were understandably enforced due to the high level of danger posed by the virus to certain groups of individuals (e.g., elderly people or people with severe vulnerabilities and/or comorbidity), some recent studies have shown that these strict policies applied to children could have caused more harm than good [2].

The international scientific community is currently engaged in a debate about this issue. However, well-established data from worldwide research have consistently shown that close, consistent, and sensitive interactions between mothers and their infants are associated with positive physiological and psychological outcomes for both parties [3]. Early skin-to-skin contact and closeness play a pivotal role in establishing the early maternal–infant attachment bond [4], crucial for adaptive child development. This contact provides essential sensory inputs, such as the mother’s voice and scent, which enhance the infant’s physiological and psychological stability and adjustment [5]. Physical separation has been found to negatively influence infants’ health and particularly impact the quality of subsequent mother–infant interactions, including feeding practices [6,7]. While genetic factors also play a role [8,9], the quality of these interactions is essential for building adaptive emotion and behavioral regulation processes in infants and serves as a protective factor against maladaptive psychological outcomes later in life [10,11,12].

Indeed, the ongoing debate surrounding the importance of parent–child (especially mother–child) closeness and intimacy is surprising. Extensive literature has demonstrated that parent–child separation should be avoided in nearly all possible cases, and authoritative scholars have penned seminal pages that seemed undisputable until the pandemic hit.

The significant contributions of Anna Freud and Dorothy Burlingam, founders of Hampstead War Nurseries for abandoned children, and the work of René Spitz and John Bowlby, who observed the early effects of mother–child separation and maternal deprivation on infantile emotional and cognitive development, underscore the crucial role of maternal care in infant development.

Spitz [12] and Bowlby [13] described children who had experienced maternal deprivation separation in the first year of life as listless and withdrawn, with reduced exploratory and relational behaviors, and as experiencing infantile mourning related to the loss of their maternal figure. Their observations on the effects of mother–child separation and maternal deprivation were pivotal in shaping the relational paradigm of psychoanalytic orientation: the theory of object relations.

Within the broad framework of the psychodynamic theory of object relations, focusing on the observational method of mother–child relationships, two converging approaches emerge.

One approach, associated with Melanie Klein, involves the development of the “Mother–Infant Observation” method [14], emphasizing the reliability of the internal mother, who is particularly capable of containing and modulating the child’s negative internal states at the onset of infantile psychic life, allowing the child to reflect and process their experiences, to think their thoughts, and to feel their feelings.

The second approach, stemming from Donald W. Winnicott’s contributions, is more focused on identifying the “reliability of the real mother”, who is a “good enough mother” with a well-tempered caregiving style, capable of gauging the quality and quantity of her interventions with her child, leading to the “reliability of the internal mother” [15,16].

Bowlby recognized the need for a new theory to explain the consequences of mother–child separation and maternal deprivation in infant development. The principle of inseparability between the child and their environment, highlighted by developments in the psychoanalytic theory of object relations and the psychology of the Self, shifted the focus of observation from the individual to the relationship, wherein both the child and the parent interact reciprocally through stimulations and shaping. This new perspective is underscored by John Bowlby’s attachment theory, which posits that children are actively and biologically pre-adapted to seek social exchanges, organizing their experiences into representations of Self and others—Internal Working Models—and necessitating appropriate responses for emotional bonds and security to foster positive development.

Standing on these giants’ shoulders, thousands of other scholars have more recently elaborated on these theories and verified them through empirical observations. However, today, we seem to overlook these lessons, for instance, by isolating children from their parents when trespassing borders illegally or in the case of sanitary emergencies.

Can we really ignore decades of uncontradicted research? We think not. This is why we believe the current literature should not relegate past contributions to the dusty shelves of abandoned libraries, but it should rather formulate new hypotheses on the robust pillars of previous scholars.
